# Flitwick Water

**Published:** 1889-07-06

**Authors:** 


### Britain's Native Resources.

### FLITWICK WATER.

Nature is believed by some persons to produce the finest medicines ready for use, and the best waters all fit for the table. But that is not always the truth. In chemistry, as in other sciences, the laboratory of man sometimes brings forth more exact, more useful, and more finished products than the laboratory of nature. Sometimes, but not always. Man can make lemonade, but he cannot make a lemon; he can produce cider, but he cannot produce an apple. Now and again, by a happy fortuity of circumstances, Nature succeeds in manufacturing in her laboratory, substances, which for beauty and utility are the despair of man, who, generation after generation, tries to imitate them in vain. What, for example, is the diamond? Pure carbon, says the chemist. But what chemist, though he may have had cart-loads of carbon at his command, ever yet succeeded in making one little real diamond?

The natural mineral waters of certain continental and British springs have all been analysed; and as far as the analysis shows there is no reason why they should not be imitated. But here is the curious fact, that the mineral waters of the laboratory, although they may yield to analysis exactly the same constituents as natural mineral waters, never have the same taste, and never seem to produce the same beneficial results. Many people are sceptical as to the value of mineral waters in general; but as a matter of fact certain mineral waters have established as secure a reputation as Homer or Shakespeare or Sir Walter Scott.

Great Britain is not very rich in mineral springs, but she has some which have already attained to a high reputation, and others which are certain to do so in time. For the last several years Flitwick, in Bedfordshire, has been emerging from obscurity, and engaging the attention both of medical men and men of commerce.

Flitwick water is a highly valuable chalybeate. It is obtained in large quantities at its native village of Flitwick. For a good many years it has had a high local reputation, although, since a prophet has little or no honour in his own country, certain local wise-acres have asked with a sceptical shake of the head, "Can any good thing come out of Flitwick?" But as a matter of fact, and notwithstanding that Flitwick has the misfortune to be a village, and a Bedfordshire village, and is principally inhabited by what are vulgarly known as "chaw-bacons," it has produced a substance of great medicinal value in its ferruginous water. The writer has had practical experience of the Flitwick water for several years, and so far as its medicinal effects are concerned he speaks from his own knowledge.

The carbonate of iron is perhaps the most widely digestible and useful of all the forms of the drug. So much is this the case that iron when given in other forms than the carbonate is sometimes combined with the carbonate of another substance, such as potassium, for example, in order that there may be a decomposition of the two substances in the stomach, and a recomposition of carbonate of iron. Of course, when the carbonate can be administered already formed, there is no necessity for such analysis and synthesis in the patient's stomach. Flitwick water contains iron in the form of carbonate, and that in very large proportion, as much, in fact, as 144 grains to the gallon. But, in addition, it contains the sulphates of magnesia and soda in considerable quantities, together with the muriate and carbonate of magnesia, and, oddly enough, certain vegetable acids, all of which have decidedly medicinal properties. It is therefore a chalybeate water of the greatest possible value: some physicians indeed affirming that it has not its equal among any of the known ferruginous waters. If it be asked when and by whom such a water should be taken, the answer is that it may be taken by almost anybody—man, woman, or child, and in almost any circumstances when iron is needed. Of course, like any other drug, iron should be taken under the direction of a competent medical man. All medical men who have not as yet had any experience of the Flitwick water, will do well to try it. Messrs. Ingram and Royle, of Farringdon-street, have the water in hand, and offer all needed facilities for prolonged experimentation.

